# Supplementary material for: Molecular Characterization of Feline Parvovirus from Domestic Cats in Henan Province, China from 2020 to 2022
Source: Vet Sci. 2024 Jun 30;11(7):292. doi: 10.3390/vetsci11070292 (PMC11281718; doi:10.3390/vetsci11070292)
Supplement: Supplementary file 1 [file vetsci-11-00292-s001.zip › Supplementary files/Table S1.DOCX]

| **Origin** | **Sampling No.** | **No. of positive** | **No. of VP2 gene detection** | **No. of NS1 gene detection** |
| --- | --- | --- | --- | --- |
| Zhengzhou | 30 | 8 | 4 | 8 |
| Luoyang | 28 | 8 | 5 | 7 |
| Xinxiang | 8 | 4 | 1 | 3 |
| Anyang | 6 | 2 | 1 | 2 |
| Shangqiu | 5 | 2 | 0 | 1 |
| Xinyang | 5 | 1 | 0 | 1 |
| Sum | 82 | 25 | 11 | 21 |

Table S1. Sample source and amplification.
