# Supplementary material for: Molecular Characterization of Feline Parvovirus from Domestic Cats in Henan Province, China from 2020 to 2022
Source: Vet Sci. 2024 Jun 30;11(7):292. doi: 10.3390/vetsci11070292 (PMC11281718; doi:10.3390/vetsci11070292)
Supplement: Supplementary file 1 [file vetsci-11-00292-s001.zip › Supplementary files/Table S2.DOCX]

**Table S1**. Refer to the sequence information of FPV *VP2* gene.

| **Strain name** | **Isolation year** | **Country/Region** | **Login ID** |
| --- | --- | --- | --- |
| FPV | 2018 | Beijing | MK266797.1 |
| FPV | 2019 | Beijing | MT270581.1 |
| FPV | 2018 | Harbin | MK671179.1 |
| FPV | 2005 | Changchun | DQ099431.1 |
| FPV | 2008 | South Korea | HQ184195.1 |
| FPV | 2006 | Italy | EU498706.1 |
| FPV | 2014 | Portugal | KT240136.1 |
| FPV | 2007 | Argentina | EU018143.1 |
| FPV | 2008 | South Korea | HQ184197.1 |
| FPV | 2013 | USA | KJ813893.1 |
| FPV | 2009 | Japan | AB000050.1 |
| FPV | 1975 | Japan | AB000066.1 |
| FPV | 1998 | Taiwan | AF015223.1 |
| FPV | 1990 | USA | JN867594.1 |
| FPV (vaccine) | 1990 | New York | M38246.1 |
| FPV (vaccine) | 2008 | Italy | EU498681.1 |
| FPV | 1978 | USA | JN867595.1 |
| FPV | 2012 | USA | KJ813895.1 |
| FPV | 2011 | USA | JX475256.1 |
| FPV | 2019 | Beijing | MT270585.1 |
| FPV | 2008 | South Korea | HQ184189.1 |
| FPV | 2018 | Tianjin | MK266794.1 |
| FPV | 2012 | Guangdong | KC473946.1 |
| FPV | 2006 | Jilin | DQ474236.1 |
| FPV | 2016 | China | MF541140.1 |
| FPV | 2019 | Italy | MT274378.1 |
| FPV | 2018 | Jilin | MK671181.1 |
| FPV | 2018 | China | MH329286.1 |
| FPV | 2017 | Harbin | MK671163.1 |
| FPV | 2017 | Guiyang | MK266788.1 |
| FPV | 2019 | China | MN419004.1 |
| FPV | 2020 | China | MW659466.1 |
| CPV (vaccine) | 2007 | South Korea | FJ197846.1 |
| CPV (vaccine) | 2008 | Taiwan | FJ011098.1 |
| CPV (vaccine) | 1990 | USA | M38245.1 |
| CPV (vaccine) | 2007 | South Korea | FJ197847.1 |
| CPV | 2009 | Argentina | JF414821.1 |
| CPV | 2010 | Uruguay | KC196095.1 |
| CPV | 2009 | USA | JN867603.1 |
| CPV | 2005 | Italy | FJ005265.1 |
| CPV | 2018 | India | MH545963.1 |
| CPV | 2014 | China | KR002805.1 |
| CPV | 2019 | China | MN473463.1 |
| CPV | 2019 | China | MN473464.1 |
