# Supplementary material for: Molecular Characterization of Feline Parvovirus from Domestic Cats in Henan Province, China from 2020 to 2022
Source: Vet Sci. 2024 Jun 30;11(7):292. doi: 10.3390/vetsci11070292 (PMC11281718; doi:10.3390/vetsci11070292)
Supplement: Supplementary file 1 [file vetsci-11-00292-s001.zip › Supplementary files/Table S3.DOCX]

**Table S2**. Refer to the sequence information of FPV *NS1* gene.

| **Strain name** | **Isolation year** | **Country/Region** | **Login ID** |
| --- | --- | --- | --- |
| FPV | 2014 | Harbin | KP280068.1 |
| FPV | 2007 | XinJiang | EF988660.1 |
| FPV | 2015 | Canada | MF069445.1 |
| FPV | 2016 | Canada | MF069447.1 |
| FPV | 2008 | USA | EU659114.1 |
| FPV | 2008 | USA | EU659113.1 |
| FPV | 2008 | USA | EU659115.1 |
| FPV | 2019 | Canada | MN862748.1 |
| FPV | 2019 | Canada | MN862749.1 |
| FPV | 2019 | Canada | MN862746.1 |
| FPV | 2019 | Canada | MN862747.1 |
| FPV | 2010 | Canada | MF069446.1 |
| FPV | 2005 | Australia | X55115.1 |
| FPV | 2008 | USA | EU659112.1 |
| FPV | 2017 | Canada | MN862745.1 |
| FPV | 2015 | Italy | KX434462.1 |
| FPV | 2019 | Hefei | MT614366.1 |
| FPV | 2015 | Italy | KX434461.1 |
| FPV | 2018 | India | MH559110.1 |
| FPV | 1999 | Thailand | KP019621.2 |
| FPV | 2018 | Guilin | MG764510.1 |
| FPV | 2015 | Kunming | MG764511.1 |
| FPV | 2020 | China | MZ005633.1 |
| FPV | 2021 | Egypt | OM638043.1 |
| FPV | 2019 | China | MN908257.1 |
| FPV | 2020 | China | MW659466.1 |
| FPV | 2014 | China | MH165482.1 |
| FPV | 2017 | Australia | MZ742178.1 |
| FPV | 2017 | Australia | MZ742175.1 |
| FPV | 2018 | China | MZ836453.1 |
| CPV | 1990 | USA | M38245.1 |
| FPV | 1990 | USA | M38246.1 |
| CPV-2a | 2018 | India | MH545963.1 |
| CPV-2a | 2014 | China | KR002805.1 |
| CPV-2b | 2000 | USA | EU659119.1 |
| CPV-2b | 2017 | Japan | LC270892.1 |
| CPV-2c | 2009 | Italy | KU508407.1 |
| CPV-2c | 2011 | Uruguay | KM457125.1 |
