# Supplementary material for: Molecular Characterization of Feline Parvovirus from Domestic Cats in Henan Province, China from 2020 to 2022
Source: Vet Sci. 2024 Jun 30;11(7):292. doi: 10.3390/vetsci11070292 (PMC11281718; doi:10.3390/vetsci11070292)
Supplement: Supplementary file 1 [file vetsci-11-00292-s001.zip › Supplementary files/Table S4.DOCX]

Table S4. Viral loading in each tested organ.

| Tested organs | Virus loading (copies/μg) | | | | |
| --- | --- | --- | --- | --- | --- |
|  | No. 1 | No. 2 | No. 3 | Mean | SE |
| Liver | 5.8×10^4^ | 4.0×10^4^ | 9.2×10^4^ | 6.3×10^4^ | 2.1×10^4^ |
| Spleen | 1.3×10^6^ | 1.3×10^6^ | 1.3×10^6^ | 1.3×10^6^ | 1.7×10^4^ |
| Lung | 1.2×10^6^ | 1.2×10^6^ | 1.1×10^6^ | 1.2×10^6^ | 3.3×10^4^ |
| Small intestine | 2.2×10^6^ | 2.4×10^6^ | 2.4×10^6^ | 2.3×10^6^ | 8.9×10^4^ |
| Kidney | 2.7×10^5^ | 2.6×10^5^ | 2.0×10^5^ | 2.4×10^5^ | 3.3×10^4^ |
| Muscle | 1.7×10^5^ | 9.3×10^4^ | 1.0×10^5^ | 1.2×10^5^ | 3.4×10^4^ |
| Stomach | 1.8×10^6^ | 1.6×10^6^ | 1.6×10^6^ | 1.7×10^6^ | 1.0×10^5^ |
| Brain | 5.1×10^4^ | 4.4×10^4^ | 4.1×10^4^ | 4.6×10^4^ | 4.2×10^3^ |
