# Supplementary figures and images for: Molecular Characterization of Feline Parvovirus from Domestic Cats in Henan Province, China from 2020 to 2022
Source: Vet Sci. 2024 Jun 30;11(7):292. doi: 10.3390/vetsci11070292 (PMC11281718; doi:10.3390/vetsci11070292)

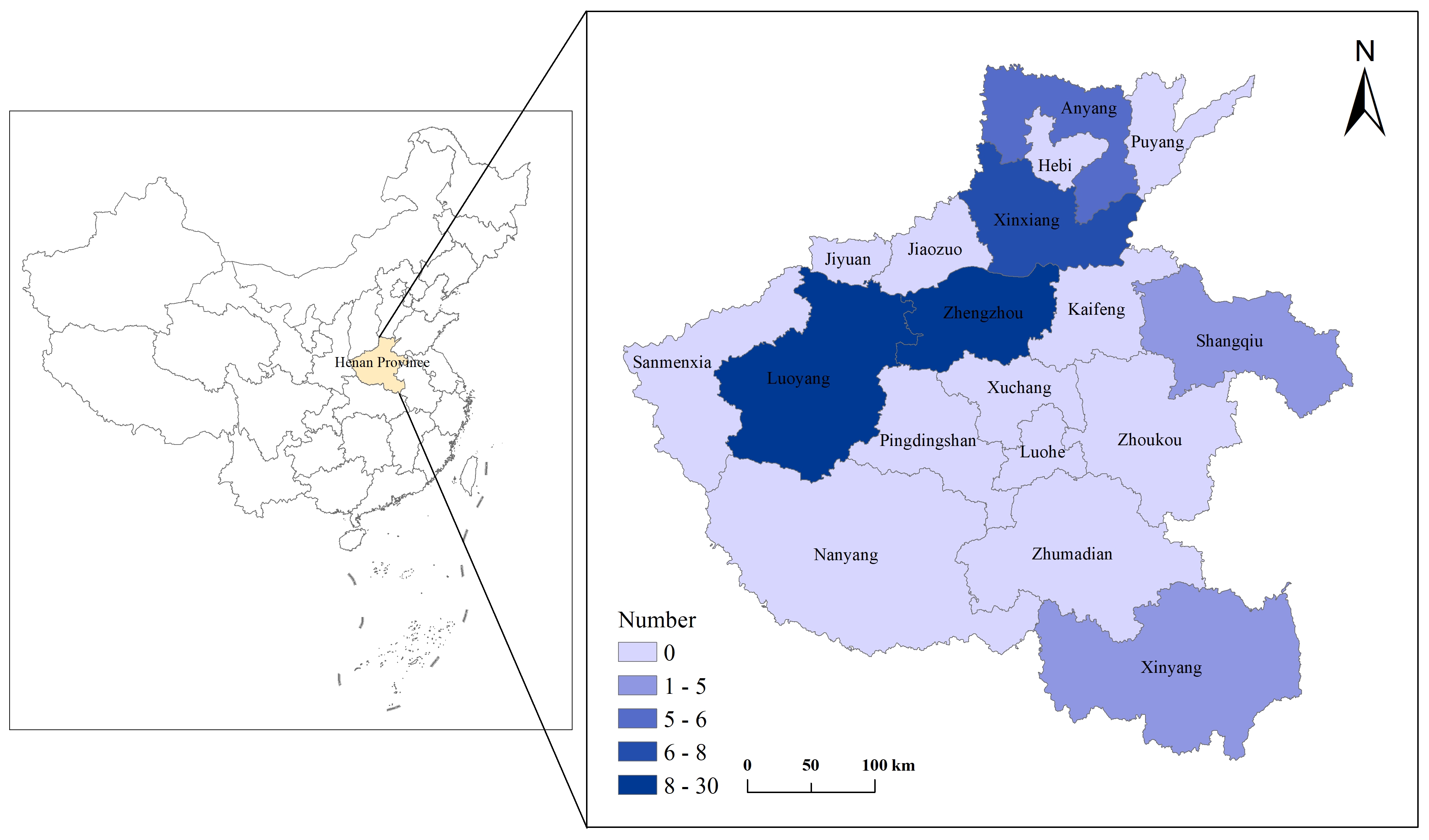

Supplement: Supplementary file 1 [file vetsci-11-00292-s001.zip › Supplementary files/Figure S1.jpg]

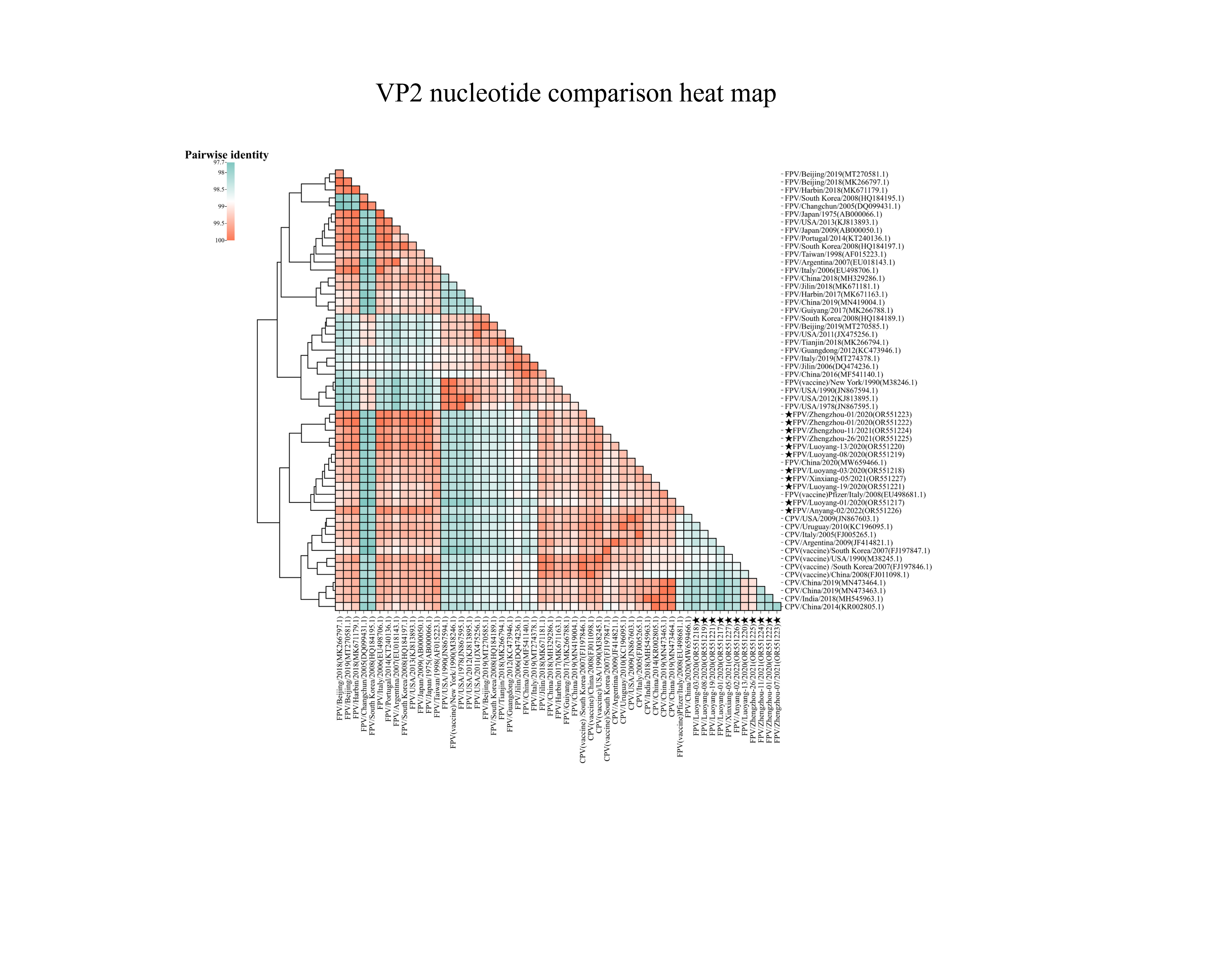

Supplement: Supplementary file 1 [file vetsci-11-00292-s001.zip › Supplementary files/Figure S2.tif]

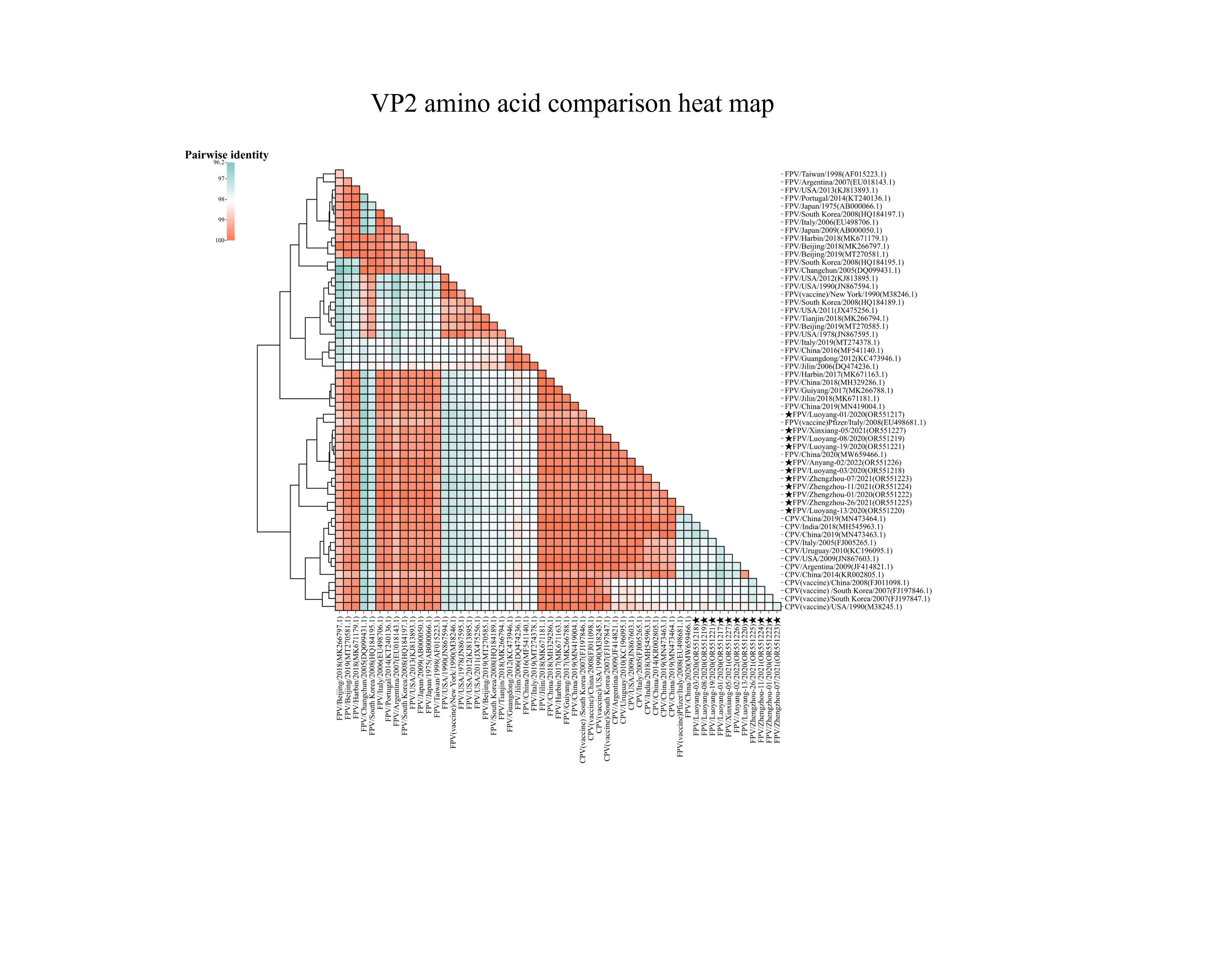

Supplement: Supplementary file 1 [file vetsci-11-00292-s001.zip › Supplementary files/Figure S3.tif]

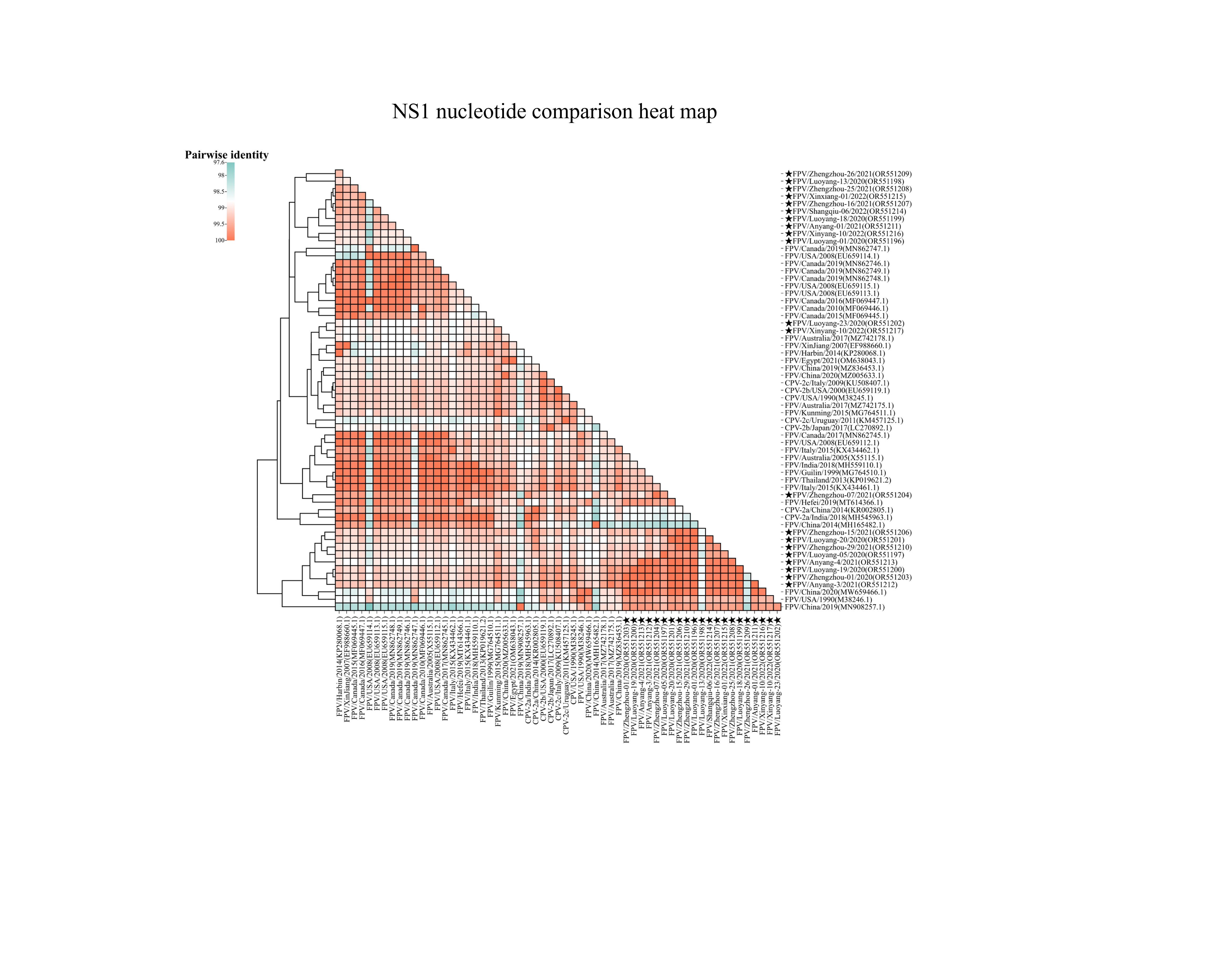

Supplement: Supplementary file 1 [file vetsci-11-00292-s001.zip › Supplementary files/Figure S4.tif]

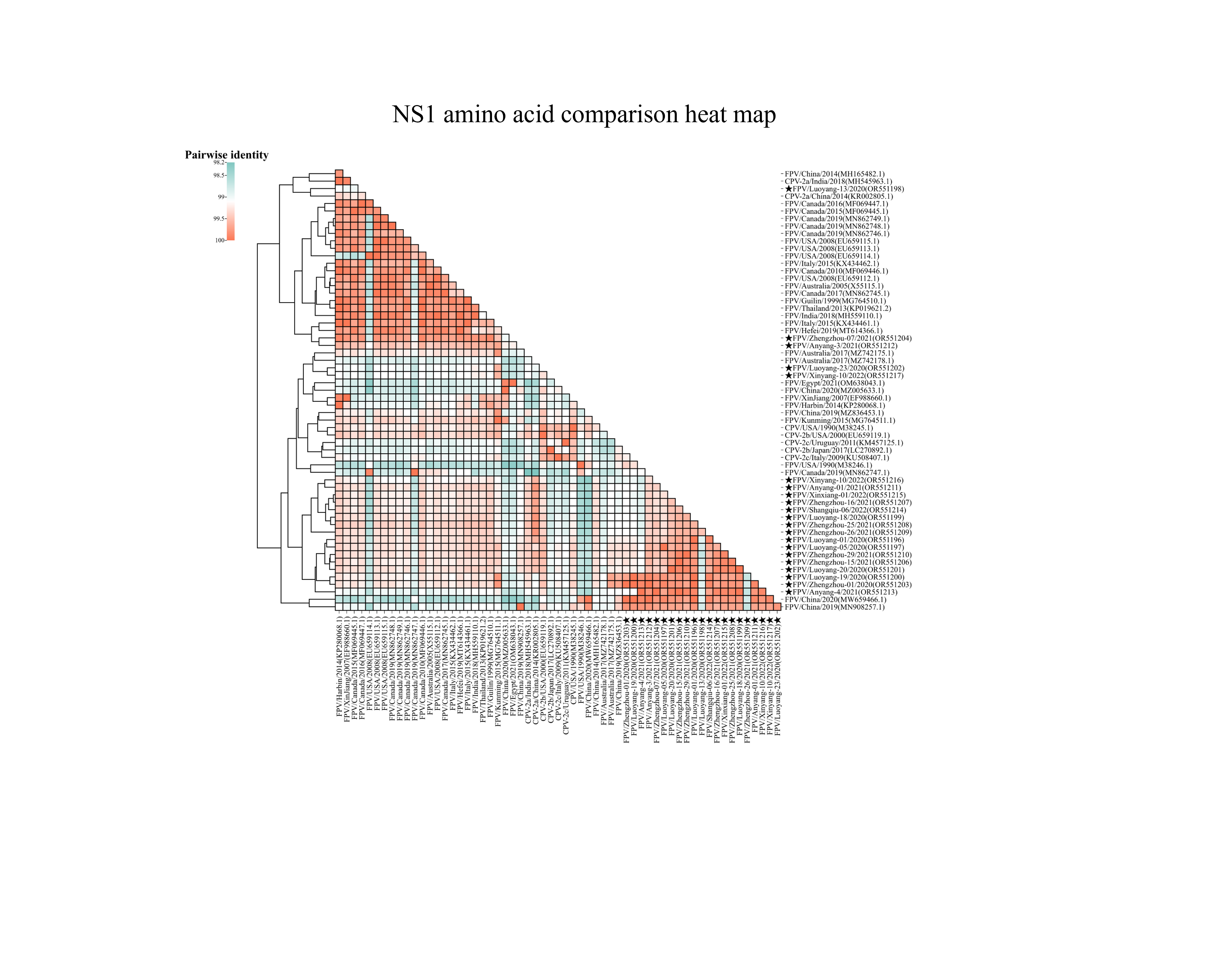

Supplement: Supplementary file 1 [file vetsci-11-00292-s001.zip › Supplementary files/Figure S5.tif]
